# Reconstructing phylogeny from metabolic substrate-product relationships

**DOI:** 10.1186/1471-2105-12-S1-S27

**Published:** 2011-02-15

**Authors:** Che-Wei Chang, Ping-Chiang Lyu, Masanori Arita

**Affiliations:** 1 Institute of Bioinformatics and Structural Biology, National Tsing Hua University, 101, Section 2 Kuang Fu Road, 30013, Taiwan; 2 Department of Biophysics and Biochemistry, Graduate School of Science, The University of Tokyo, Bunkyo-ku Hongo 7-3-1, 113-0033 Tokyo, Japan; 3 Computational Biology Research Center, National Institute of Advanced Industrial Science and Technology, Koto-ku Aomi 2-42, 135-0064 Tokyo, Japan; 4 Institute for Advanced Biosciences, Keio University, Baba-cho 14-1, Tsuruoka City, 997-0035 Yamagata, Japan

## Abstract

**Abstract:**

**Background**

Many approaches utilize metabolic pathway information to reconstruct the phyletic tree of fully sequenced organisms, but how metabolic networks can add information to original genomic annotations has remained open.

**Methods**

We translated enzyme reactions assigned in 1075 organisms into substrate-product relationships to represent the metabolic information at a finer resolution than enzymes and compounds. Each organism was represented as a vector of substrate-product relationships and the phyletic tree was reconstructed by a simple hierarchical method. Obtained results were compared with several other approaches that use genome information and network properties.

**Results**

Phyletic trees without consideration of network properties can already extract organisms in anomalous environments. This efficient method can add insights to traditional genome-based phylogenetic reconstruction.

**Conclusions**

Structural relationship among metabolites can highlight parasitic or symbiont species such as spirochaete and clamydia. The method assists understanding of species-environment interaction when used in combination with traditional phylogenetic methods.

## Background

Understanding the phyletic relationship among living organisms has long been a fundamental challenge since the concept of evolution had emerged. Traditionally, molecular biologists constructed phylogenetic trees based on the sequence similarity of small subunit ribosomal RNA [1] or other single genes. As whole-genome sequencing technologies advance, vast amount of sequence data become available for download and analysis. Without question, the comparative analysis of whole genomes can provide more information to reconstruct the phylogeny than individual genes do. Consequently, numerous methods have been proposed to reconstruct the phylogenetic trees from whole genome features such as oligonucleotide compositions [2], genome fragment occurrence [3], and absence/presence of metabolic features [4].

In parallel with genomic comparisons, many studies focused on the similarity of metabolic processes. Metabolic profiles of a living organism are strongly related to its environment, and metabolism is adapted to balance compounds taken up from its surroundings [5,6]. Thus, metabolic consideration can add insights into species-environment interaction such as symbiosis or convergent adaptation to extreme environments. To analyze the phyletic relationship in metabolic capability, there are at least 3 approaches. The first is machine learning. Oh *et al.* used a distance computed by the exponential graph kernel, i.e., the weighted sum of similarities between adjacency matrices of 1-step neighbors, 2-step neighbors, and so on for 81 organisms [7]. The second is network comparison. Zhang *et al.* defined existence/absence of metabolic pathways and computed the network similarity measure for 47 organisms [8]. The last is EC-based classification. Clemente *et al*. used sets of EC numbers to define pathway similarity and compared metabolism of 8 bacteria [9].

Metabolic data are well standardized in previous approaches because all works depended on the bulk-downloadable KEGG database [10]. Less concerned, however, was the strategy for transforming enzymatic reactions into graphs (or networks). Depending on the strategy, resulting networks are drastically different enough to change fundamental network centralities [11]. For example, Borenstein *et al.* converted each enzymatic reaction to a fully connected bipartite graph between substrates and products to enhance connectivity and defined ‘seed’ compounds for each organism as the union of essential metabolites in all environments [12]. This transformation is known to overestimate the ability to synthesize/degrade metabolites. On the other hand, using the EC numbers for pathway analysis tend to underestimate the metabolic network because the numbers are assigned to biochemical transformation, and not to enzyme themselves. We here propose a more suitable data representation, and elucidate the phylogenies across three domains of life. Its effectiveness is shown in comparison with previous approaches.

## Methods

### Enzyme annotation for organisms

Enzyme annotations and corresponding EC reactions for 1075 organisms (895 bacteria, 67 archaea, and 113 eukaryotes) were obtained from the KEGG database through its application program interface. The number of EC reactions was 3116, covering as many as 154 pathway maps. Metabolic annotations in each species were represented as a set of substrate-product relationships by transforming all assigned EC reactions into a set of metabolite pairs (see the next section). Most EC-numbered entries correspond to multiple enzymatic reactions. For example, alcohol dehydrogenase (EC 1.1.1.1) can catalyze a multitude of compounds with a hydroxyl group. For such generic EC-numbered functions we manually integrated possible reactions to ensure the coverage of biochemical transformation shown in the metabolic maps.

### Strategy for graph transformation

An enzymatic reaction usually has multiple inputs (*substrates*) and outputs (*products*). Although standard metabolic pathway charts are depicted as hypergraphs, substrate-product relationships must be specified for each reaction to transform it into a graph. A standard way is to use a fully connected bipartite graph [7,8,12]. The network connectivity then portrays the ‘reaction membership’; frequently occurring metabolites become hub nodes in the resulting graph. The representation, however, does not capture biochemical transformation between compounds because any two metabolites can be falsely linked through metabolic hubs regardless of their structures [11].

To avoid this bypassing effect, we employ the substrate-product decomposition of reactions [13]. In this scheme, each reaction is decomposed into a set of structurally related substrate-product pairs at the atomic scale. The data are also available from the RPAIR database [14], and the same method has been used in several recent works [15-17]. This representation avoids bias originating from currency metabolites. In other words, the method focuses on the variation of structural transformations, not the occurrence of each metabolite. The decomposition results of EC-numbered reactions are accessible at our wiki-based site: http://metabolomics.jp/wiki/Enzyme:[EC-number]. For example, the details of hexokinase can be accessed at http://metabolomics.jp/wiki/Enzyme:2.7.1.1 In the transformation, we replaced generic names such as alcohol or amino acids with concrete compound names. For hexokinase, as many as 15 reactions are included depending on hexose types. Through this decomposition, a set of enzymatic reactions becomes a set of substrate-product pairs. We did not consider the multiplicity of each pair in our analysis.

### Phyletic reconstruction

Phyletic trees were created by a hierarchical clustering method (pairwise complete linkage algorithm) using the Cluster 3.0 software program [18]. Each organism was represented as a vector of substrate-product pairs, where the absence/presence of each relationship was denoted as 0 or 1. For visualization, Dendroscope software program [19] was used to analyze and compare phyletic trees. The employed simple algorithm may be controversial for phyletic reconstruction, and will be discussed later.

## Results

We compared results of our data representation with several recent, well known studies.

### Phyletic trees for multi-domains of life based on substrate-product relationships

**Figure 1:**
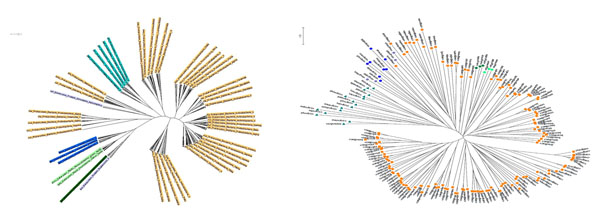
**Reconstructed phlogeny**. Left: Our reconstruction. Species in the same family were grouped into leaf nodes. Right: Reconstruction by Borenstein *et al*. [12] using the ‘seed’ metabolites. Reprinted with permission. Copyright (2008) National Academy of Sciences, U.S.A. Abbreviations: Bac, bacteria (orange); Arc, archaea (cyan); Pla, plants (light green); Ani, animals (navy blue); Fun, fungi (dark green); Pro, protists (light purple).

To compare with the phylogeny reconstruction based on the ‘seed’ metabolites [12], we reconstructed a phyletic tree for 478 species (the same number as the original article) using our substrate-product pairs. Figure 1 shows the summarized view of both trees of life. Both approaches clustered 6 main domains successfully, but the seed approach placed plants and fungi among bacteria. This is a serious artifact; since the seed approach focuses on essential metabolites, classification based on secondary metabolites becomes unstable. In both trees, a few seemingly dispersed clades (protists in eukaryota) existed. This is reasonable because the definition of protist is a structural simplicity regardless of its metabolic capability. Note here that our method correctly classified eukaryotes and also placed spirochaeta and chlamydia in a group separated from the other bacteria. This indicates these parasitic/pathogenic species exhibit anomalous metabolism in comparison with the other species, but further investigation is necessary to confirm its reason.

**Figure 2:**
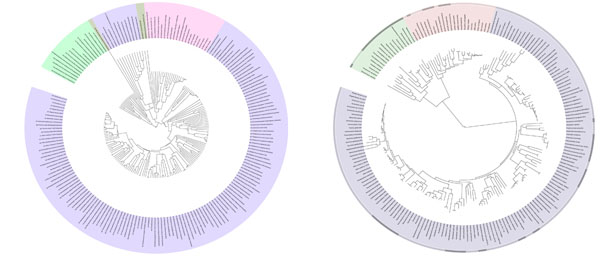
**Reconstructed phylogeny**. Left: Our reconstruction. Right: Reconstruction by Ciccarelli *et al*. [20] using the 31 universal protein families. Reprinted with permission from AAAS. Bacteria (purple), Archaea (green), thermo Archaea (yellow green), Metazoa (pink).

As the second comparison, our approach was compared with the golden standard tree, reconstructed by using concatenated alignment of 31 universal protein families covering 191 species [20] (Figure 2). Our method could clearly separate three main domains, bacteria, archaea and metazoan, except *Nanoarchaeum equitans*, which is an obligatory symbiont on *Ignicoccus*. It lacks many essential metabolic pathways and therefore became an orphan branch in our reconstruction. Similarly, the reconstruction reflected more on metabolic phenotypes rather than genetic evolution. For example, *Mycoplasma spp*. were located far from the other bacteria and closer to eukaryotes in our tree because they lack many metabolic pathways (higher animals lack many amino acid biosynthesis, for example). This defect was also observed in the comparison with the ‘seed’-based tree. Some invertebrate parasites were also grouped with *Caenorhabditis elegans* due to their metabolic similarity of unknown reason. Note that systematics of *C. elegans* is contentious and still unresolved because of its high evolutionary rate [21]. In summary, our method could reproduce comparable results with the standard tree. In addition, it could extract metabolically anomalous species which could not be easily found by simple genetic comparisons by comparing results with the standard phylogeny.

### Phyletic trees with or without network connectivity

**Figure 3:**
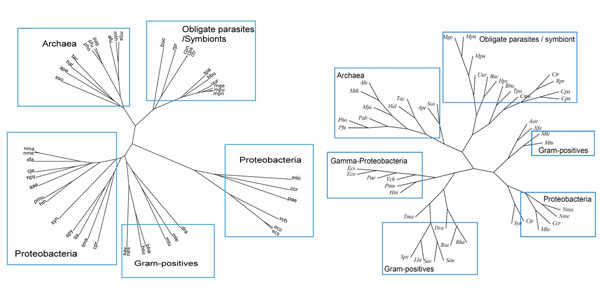
**Reconstructed phylogeny**. Left: Our reconstruction. Right: Reconstruction by Zhang *et al.*[*8*]. Reprinted under the BioMed Central Open License agreement (BMC Bioinformatics).

To investigate the information gain by considering metabolic network connectivity, we carefully compared our approach with the network topology-based approach [8]. There are few discrepancies between our and their results. In our approach, some proteobacteria and hyperthermophils were not properly grouped into the same sub-clusters (Figure 3). These clades are labeled as “other independent bacteria” and their proper positions are context-dependent. For this reason, we do not consider our classification inappropriate. On the other hand, we could correctly cluster *Mycobacterium tuberculosis* and *M. leprae* into Gram-positive bacteria. In addition, parasites and symbionts (spirochaete and clamydia) were classified more correctly in our method. In summary, although overall classification was similar, we could better, or at least equally, classify parasitic or symbiotic species in comparison with the results with another phyletic approach.

### Comparison with EC number-based classification

**Figure 4:**
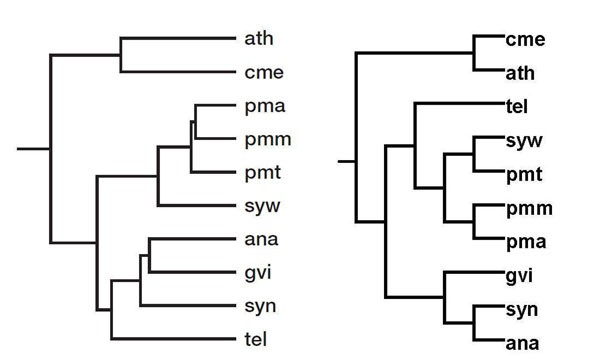
**Reconstructed phylogeny.** Left: Our reconstruction. Abbreviations: *Anabaena* (ana), *Gloeobacter violaceus* (gvi), *Prochlorococcus marinus marinus* (pma), *P. marinus pastoris* (pmm), *P. marinus* (pmt), *Synechocystis* (syn), *Synechococcus* (syw), *Thermosynechococcus elongatus* (tel). Most separated are the two photosynthetic eukaryotes: *Arabidopsis thaliana* (ath) and *Cyanidioschyzon merolae* (cme). Right: EC-number based classification by Clemente *et al*. [9] Reprinted with permission from Oxford University Press.

Clemente *et al*. investigated the relationship among 8 photosynthetic bacteria using pseudo-alignment of over 60 metabolic pathways using the EC hierarchy [9]. Lastly, we compared their results with ours and found 2 differences from the EC-based phylogeny (Figure 4): the positions of *Synechocystis* (syn) and *Synechococcus* (syw), both of which belong to Chroococcales together with *Thermosynechococcus elongates* (tel). The misplacement of Chroococcales was observed in the work by Clemente *et al.* too and presumably results from the insufficiency of gene annotations in these species (Figure 4). In terms of metabolic similarity, our reconstruction seems more accurate because *Gloeobacter violaceus* (gvi) and tel were isolated from rocks and hot springs, respectively, whereas the remaining 6 species were isolated from fresh or sea water. Therefore, the two species should be regarded as metabolic out-groups as in our classification.

### Central metabolites

We previously argued that metabolic hubs are better identified in the substrate-product graph than in other graph representations, because the approach does not count the frequency of metabolite names in reactions but the number of structural transformations [11]. The number of transformations roughly reflects the structural variation of catalytic sites of respective enzymes, and therefore reflects the diversity of metabolic capabilities.

Table 1 is the list of metabolites in the three domains which appear as the top 10 hubs in more than 20% organisms for each domain. The abundance of adenosine-related metabolites for all domains indicates the ancientry of purine-related metabolism, which coincides with the analysis on protein structures [22]. The presence of CO_2_ and NH_3_ is an unavoidable artifact of counting all decarboxylations and amino-transfers. High-degree metabolites are largely conserved. It can be seen that eukaryotes contain more reactions with glucuronate, glutathione, and galactose, which appear in drug metabolism. At the same time, eukaryotes use less L-aspartate- or 5-phospho-alpha-D-ribose 1-phosphate-dependent reactions. Archaea lack malonyl-acyl carrier proteins and coenzyme A, which often appear in lipid metabolism for eukaryotes and bacteria. Archaea also use L-glutamine more often than the other domains.

**Table 1:** Most differently transforming metabolites in the three domains. The full list is available at http://sarst.life.nthu.edu.tw/metabolic/SD.csv.

Domain	List of hubs in the descending order of appearance
Archaea (67 spp)	ATP, **L-Glutamate**, CO_2_, Acetyl-CoA, **L -Glutamine**, Pyruvate, NH_3_, L -Aspartate, AMP, 5P-alpha-D-ribose 1P, *S*-Adenosyl- L -methionine
Bacteria (895 spp)	CO_2_, ATP, NH_3_, Pyruvate, L -Glutamate, Acetyl-CoA, 5P-alpha-D-ribose 1P, **CoA, Malonyl-ACP**, L -Aspartate, Glutathione, AMP, *S*-Adenosyl- L -methionine, Glycine
Eukaryotes (113 spp)	L -Glutamate, CO_2_, Acetyl-CoA, NH_3_, **CoA**, ATP, AMP, **Glutathione**, Pyruvate, **Malonyl-ACP**, *S*-Adenosyl- L -methionine, Glycine, **D-Galactose, UDP-glucuronate**, L -Serine, L -Glutamine

Highlighted metabolites are mentioned in the main text. Abbreviations: P … phosphate; ACP … acyl carrier proteins.

### Metabolic differences between bacteria, archaea, and eukaryotes

**Figure 5:**
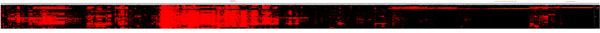
**The heat map of substrate-product relationships in 535 organisms**. The horizontal line at the topmost right part corresponds to animal- and plant-specific pathways (the rightmost line is plants. Animals are the next-right line just below plants. The black horizontal line just below the eukaryotes (plants and animals) is Mycoplasma, which lack most pathways. Archaea are clustered at the bottom of the figure.

To elucidate the metabolic differences between the three domains of life, we created a heat map of the substrate-product relationships in 535 species. In Figure 5, the vertical and horizontal directions are the hierarchically clustered organisms and the substrate-product relationships, respectively. Note that substrate-product relationships in species-specific pathways tend to cluster in this scheme. Archaea and Mycoplasma lack the fatty acid biosynthesis and many other pathways. However, many archaeal pathways are overlooked in the KEGG annotations (e.g. energy metabolism and ether-lipid metabolism for membrane synthesis), and their uniqueness is not easily discerned in this analysis. In contrast, Plantae and Animalia kingdoms in eukaryotes are easy to locate because animals possess drug- and other secondary metabolism, and plants possess unique secondary biosynthetic pathways (Figure 5).

## Discussion

Our reconstruction using substrate-product relationships efficiently extracted metabolically interesting species in comparison with the standard phylogenetic approach. Previous approaches which used metabolic information could also produce informative results [7-9,12], but the achievements were similar to those found by genetic comparisons [2-4]. This is understandable because in their approach metabolic reactions correspond roughly one-to-one to enzymes or genes.

### Why can substrate-product relationships add insights?

Our approach is more robust to pathway gaps (incomplete annotation) or currency metabolites by evaluating each biochemical transformation with an equal weight. It is also robust to biases by the number of genes or their multiplicity. Standard phylogenetic methods can elucidate evolutionary relationship, whereas our approach can locate species of anomalous or interesting metabolism in comparison. Therefore, the method is useful in combination (not exclusive) with existing phyletic/phylogenetic clustering.

Our method is also computationally lightweight and scalable, requiring O(*N^2^V*) time for computing pairwise similarity, where *N* is the number of organisms and *V* is the maximum number of reactions in one organism. On the contrary, for example, the exponential graph kernel requires O(*NV*^3^+*N*^2^*V*^2^) time to compute the similarity [7]. Our computational complexity is equivalent to the recently presented pathway alignment method [23], but the method exploits the graph topology and the result is expected to be similar to the one by Zhang *et al*[8]. Lastly, the ‘seed’ approach uses a heuristic to find metabolic seeds [12], but an accurate identification of metabolic seeds is NP-complete [24]. There is a huge gap as to the scalability to the other metabolic approaches.

### Algorithms to find phylogeny

Our method uses a simplistic complete linkage clustering algorithm to reconstruct the phylogeny. This may sound inappropriate but is grounded on our data representation. Since the substrate-product relationship disregards the occurrence of metabolites, a frequently appearing reaction type (e.g. ATP-kinase) and a rare reaction type (e.g. sterol synthase) are given the same weights. For this reason, standard parsimony or evolutionary distance does not properly reflect the distance between species in our scheme. Since we wanted to focus on metabolic differences, the complete linkage method was employed. However, other algorithms should be systematically tested and evaluated for their appropriateness, which is left as our future work.

### Sharing metabolic knowledge through wiki

We publicize the substrate-product relationships on a wiki-based site so that readers can check every detail of our analysis. This is especially important in the era of high-throughput data management because more and more research results tend to become irreproducible due to the insufficiency of publicized data or incomplete description of methods. To overcome this difficulty, the traceability and transparency of data and their analysis is important in the evaluation of research.

## Conclusions

Phylogeny was reconstructed by using structural relationship between annotated metabolites. This method is robust to pathway gaps or gene copy numbers, and can extract metabolically anomalous species by comparing the result with other phyletic or phylogenetic reconstructions. Through several comparisons, our method could highlight metabolic anomaly in clamydia and spirochaete, both of which are well known parasitic species. The metabolic comparison thus assists understanding of species-environment interaction in combination with other gene-oriented strategies.

## Competing interests

There is no competing interests.

## Authors' contributions

MA designed, and CWC conducted research under supervision of PCL. CWC and MA wrote the paper together.
